# Searching for the perfect match: can non-antibiotic antimicrobials improve bacteriophage performance?

**DOI:** 10.3389/fcimb.2026.1758422

**Published:** 2026-02-25

**Authors:** Katarzyna Kosznik-Kwaśnicka, Agnieszka Necel, Lidia Piechowicz

**Affiliations:** Department of Medical Microbiology, Faculty of Medicine, Medical University of Gdańsk, Gdansk, Poland

**Keywords:** antibiotics, antimicrobial resistance, bacteriocins, bacteriophage, essential oils, nanoparticles, phage therapy, synergistic therapy

## Abstract

The rapid spread of multidrug-resistant (MDR) bacteria worldwide has significantly reduced the effectiveness of traditional antibiotics, leading to increased interest in bacteriophages as alternative or supplementary antimicrobial agents. While phage therapy has notable benefits, such as high specificity and minimal impact on beneficial microbiota, its use alone is limited by factors like a narrow host range, quick development of resistance, complex pharmacokinetics, and challenges in delivery within biological and environmental contexts. Although combining phages with antibiotics has been shown to improve antibacterial effects, growing regulatory restrictions and efforts to minimize antibiotic use call for the exploration of non-antibiotic combination approaches. This review explores the synergistic interactions between bacteriophages and various non-antibiotic antimicrobials, including essential oils, bacteriocins, nanoparticles, and other physicochemical or host-derived agents. We present evidence that these agents can boost phage effectiveness by altering bacterial membrane integrity, stress responses, biofilm structure, and phage stability, and by delaying the emergence of resistance. Importantly, we emphasize that the observed synergies are highly context-dependent and discuss limitations related to reproducibility, safety, and translational application. Overall, this review highlights the potential of non-antibiotic compounds as tailored adjuvants to broaden the use of phage-based antimicrobial strategies in clinical, food safety, and agricultural contexts.

## 1 Introduction

The rise of infections caused by multidrug-resistant (MDR) pathogens has compromised the efficacy of conventional antibiotic treatments, posing a global threat to public health, food safety, and modern medicine (Samtiya et al., 2022; Ahmed et al., 2024; Naghavi et al., 2024). As antimicrobial resistance (AMR) spreads across both pathogenic and commensal bacterial populations, the urgency to discover alternative or complementary strategies to traditional treatment has intensified (Bassetti et al., 2009; Ahmed et al., 2024). In parallel with the increasing adoption of stewardship policies and regulatory restrictions on antibiotic use in clinical, agricultural, and food-production settings, non-antibiotic approaches are gaining renewed attention.

**Figure 1:**
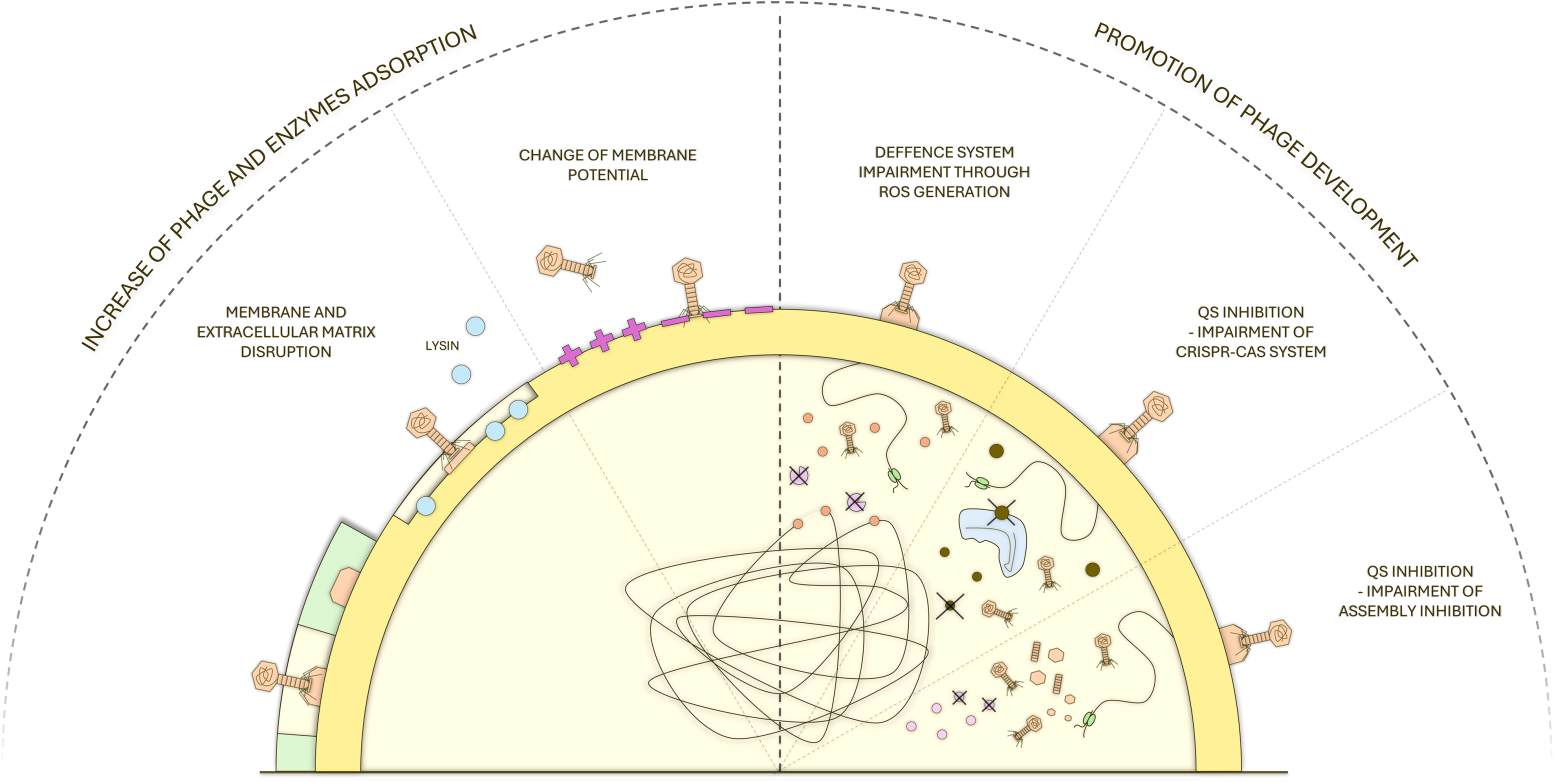
Schematic representation of proposed synergy mechanisms between bacteriophages and other antimicrobials. Non-antibiotic agents promote phage development, adsorption, and lysin activity, disrupt membranes and extracellular matrices, alter membrane potential, inhibit quorum sensing and CRISPR–Cas defenses, and induce reactive oxygen species, collectively enhancing phage infection and bacterial killing.

One such approach involves the use of bacteriophages (phages)—viruses that specifically infect bacteria. Since their discovery over a century ago, phages have been explored as therapeutic agents due to their high specificity, minimal disruption of the host microbiota, and capacity for evolutionary adaptation. However, following the introduction of penicillin and the inconsistent outcomes of early phage applications, interest in phage therapy declined in much of the Western world (Górski et al., 2019). Only a few facilities in Eastern and Central Europe studied phage therapeutic potential and decided to use them either as Over-the-Counter (OTC) medications or as an experimental therapy (Parfitt, 2005; Żaczek et al., 2022). However, interest in phage therapy has recently resurged in the West due to the AMR crisis and advances in genomics, synthetic biology, and microbiome science. While phage therapy offers unique advantages, such as high specificity, minimal disruption of the host microbiota, and evolutionary adaptability, it has limitations when used in isolation. These include narrow host range, rapid development of bacterial resistance to phages, and challenges associated with phage delivery and pharmacokinetics (Labrie et al., 2010; Lin et al., 2022; Nang et al., 2023). To overcome these limitations, it is recommended to use phage cocktails or pair phage preparations with other antimicrobials (Wright et al., 2021; Morris et al., 2025). To date, phage–antibiotic combinations have been the most extensively studied, demonstrating enhanced bacterial killing and reduced emergence of resistance to either agent (Loganathan et al., 2024; Qin et al., 2024; Morris et al., 2025). However, widespread efforts to limit antibiotic use, together with regulatory bans in certain sectors and concerns regarding toxicity, allergies, and environmental impact, restrict the long-term applicability of antibiotic-based combinations (Tang et al., 2017; Kahn et al., 2019). These limitations have stimulated growing interest in pairing bacteriophages with non-antibiotic antimicrobial compounds. Several studies have demonstrated that phage synergy with non-antibiotic antimicrobials can improve bacterial eradication, delay or prevent resistance development, and enhance biofilm penetration. Those may help through, e.g., increased efficacy of phage adsorption, interference of bacterial stress responses (Philippe et al., 2020; Lin et al., 2024), alteration of bacterial membrane integrity or cell wall charge (Alisigwe et al., 2025), and creation of oxidative environments that both impair bacterial defense systems and stabilize phage particles (Philippe et al., 2020; Alisigwe et al., 2025; Wdowiak et al., 2025b). These proposed synergy mechanisms are shown in **Figure 1**.

In this review, we examine the synergistic interactions between bacteriophages and selected antimicrobial compounds, considering mechanistic principles, practical limitations, and translational potential across clinical, food-safety, and agricultural contexts.

## 2 Limitations of phage therapy

Bacteriophage therapy has demonstrated clear therapeutic potential, with numerous case studies reporting successful treatment of pneumonia, sepsis, skin, and urinary tract infections caused by antibiotic-resistant pathogens (Valente et al., 2021; Żaczek et al., 2022). Despite these advantages, phage therapy faces several intrinsic and context-dependent limitations that restrict its widespread and predictable application. One of the most significant constraints is the narrow host range of most bacteriophages. Unlike broad-spectrum antibiotics, phages typically infect only specific bacterial species or even individual strains. This specificity arises from precise interactions between phage receptor-binding proteins and bacterial surface structures, meaning that even minor alterations in receptors can abolish phage infectivity (Hyman and Abedon, 2010; Holtappels et al., 2023; Chung et al., 2023). While high specificity is beneficial for preserving the commensal microbiota, it complicates clinical use, as pathogens must often be isolated and characterized prior to treatment. This requirement can delay therapy, particularly in acute or life-threatening infections, and limits the immediate applicability of standardized phage preparations.

**Figure 2:**
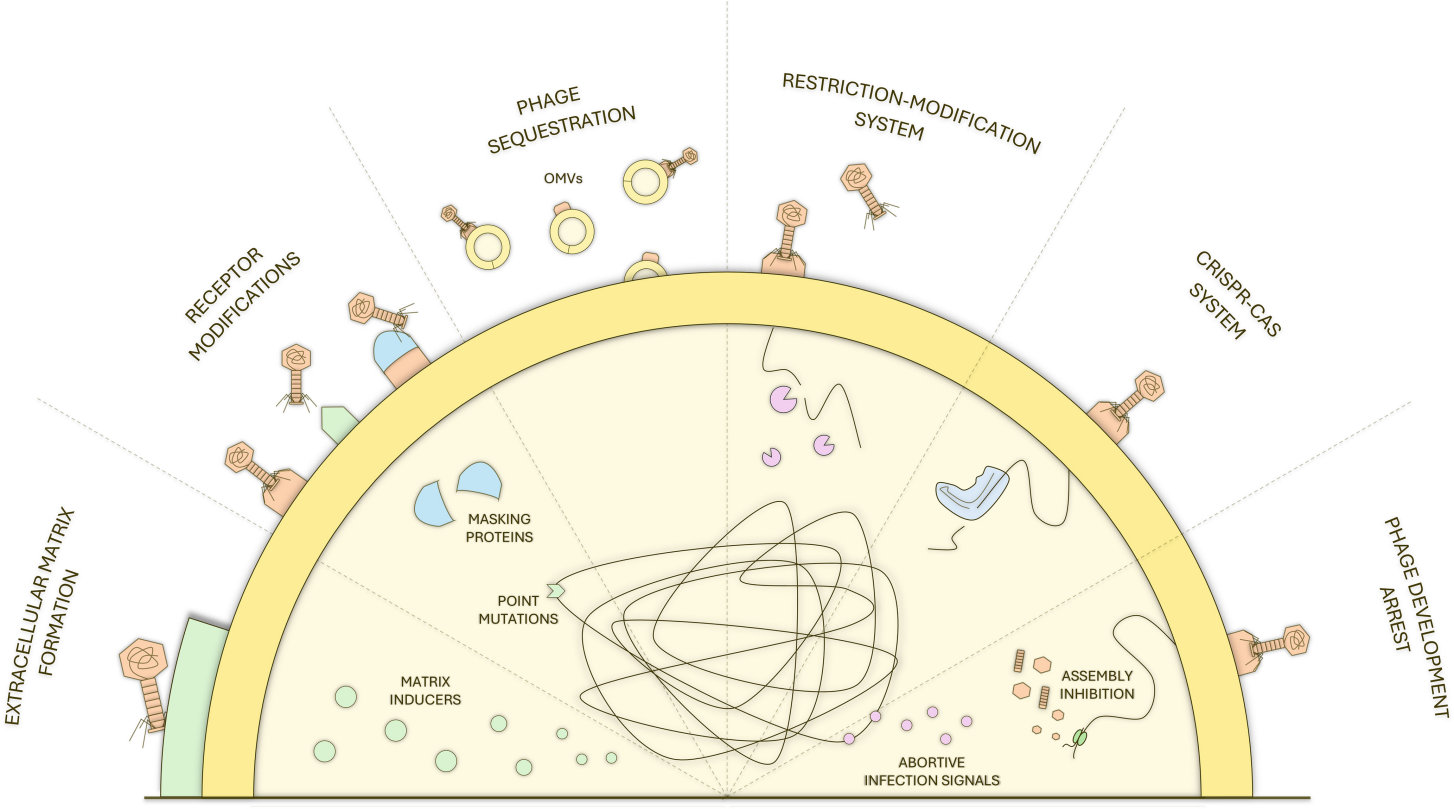
Schematic illustration of bacterial anti-phage defense mechanisms. These include receptor modification and phage sequestration by outer membrane vesicles, restriction–modification and CRISPR–Cas systems targeting phage genomes, inhibition of phage development and assembly, abortive infection signaling, and extracellular matrix formation, collectively limiting phage adsorption, replication, and propagation.

Another major limitation is the emergence of bacterial resistance to bacteriophages. Bacteria may evade phage infection through diverse mechanisms, including receptor modification or loss, CRISPR–Cas systems, restriction–modification systems, and abortive infection pathways (**Figure 2**) (Oechslin, 2018; Oromí-Bosch et al., 2023). Although it has been shown that phages coevolve with their hosts and that new phages can be easily isolated from the environment, the emergence of phage resistance remains a major concern (Koskella and Brockhurst, 2014; Jurczak-Kurek et al., 2016; Batinovic et al., 2019; Egido et al., 2022). While the use of phage cocktails can slow down the emergence of phage-resistant bacteria, they increase the complexity of the therapeutic and pose a regulatory burden on the use of phage-based products (Oromí-Bosch et al., 2023; Faltus, 2024). On the other hand, many resistance mechanisms impose physiological or fitness costs on bacteria, altering surface structures, metabolic activity, or stress responses. These trade-offs may create vulnerabilities that can potentially be exploited by complementary antimicrobial agents. Beyond phage–bacteria interactions, host-related factors also influence phage therapy outcomes. Phages introduced into the human or animal body can be recognized and neutralized by the immune system, particularly after repeated administrations. Components of both innate and adaptive immunity, like macrophages, complement proteins, and phage-specific antibodies, may reduce the administered phage titer before it reaches its bacterial targets (Górski et al., 2012; Podlacha et al., 2021; Łusiak-Szelachowska et al., 2022; Champagne-Jorgensen et al., 2023; Gembara and Dąbrowska, 2024). In addition, the pharmacokinetics and pharmacodynamics of phages are more complex than those of conventional antimicrobials, as phage concentrations at infection sites depend on bacterial titer, replication dynamics, route of administration, and host immune responses (Chatain-Ly, 2014; Nang et al., 2023). Effective phage delivery can be further hindered by physical and environmental barriers. Biofilms, intracellular niches, mucus layers, poorly vascularized tissues, and complex matrices such as food or plant surfaces can limit phage access to bacterial cells and reduce therapeutic efficacy (Barr et al., 2015; Bichet et al., 2021; Chang et al., 2022). Although phages can, in some contexts, penetrate biofilms or interact with eukaryotic cells, their activity in such environments is often inconsistent and highly context-dependent.

Collectively, these limitations highlight that the success of phage therapy is governed not only by phage–host specificity, but also by bacterial physiology, resistance dynamics, host immunity, and environmental constraints. These challenges have driven growing interest in rational combination strategies designed to support phage activity, improve delivery, and suppress resistance, rather than relying on phages as stand-alone antimicrobials.

## 3 Phage-antibiotic synergy as a conceptual framework

Combination therapies pairing bacteriophages with antibiotics, commonly referred to as phage–antibiotic synergy (PAS), have been extensively investigated as a strategy to enhance antibacterial efficacy and limit the development of resistance. Numerous *in vitro* and *in vivo* studies have demonstrated that carefully selected phage–antibiotic combinations can outperform either agent alone, leading to improved bacterial removal and, in some cases, halt the emergence of resistant subpopulations (Gu Liu et al., 2020; Zhao et al., 2024; Loganathan et al., 2024). Mechanistically, PAS arises through several processes. Antibiotics that interfere with bacterial cell wall synthesis or DNA replication can induce physiological changes, such as filamentation, altered metabolism, or stress responses, that increase susceptibility to phage infection and replication (O’Rourke et al., 2020; Lee et al., 2025). In some systems, sublethal antibiotic concentrations have been shown to enhance phage adsorption, accelerate intracellular phage production, or increase burst size, amplifying antibacterial effect (Comeau et al., 2007; Knezevic et al., 2021; Loganathan et al., 2024). Conversely, phage-induced bacterial lysis may increase antibiotic penetration or expose previously inaccessible subpopulations of bacteria (De Soir et al., 2025). Importantly, PAS can also influence resistance dynamics. Antibiotic pressure may limit the emergence of phage-resistant mutants, while phage presence in the culture can suppress antibiotic-resistant bacterial subpopulations or impose fitness costs that restore antibiotic sensitivity (Oechslin, 2018; Qin et al., 2024). These effects highlight that synergy is not merely additive, but is based on complex interactions between bacterial physiology, stress responses, and evolutionary trade-offs (Mangalea and Duerkop, 2020; Zhang and Ahn, 2025).

Despite these advantages, the clinical and industrial applicability of PAS is limited by efforts to reduce the use of antibiotics. Global antibiotic stewardship programs, regulatory restrictions in agriculture and food production, and concerns about toxicity, allergic reactions, and environmental dissemination limit the feasibility of antibiotic-based combination strategies (Tang et al., 2017; Kahn et al., 2019).

**Figure 3:**
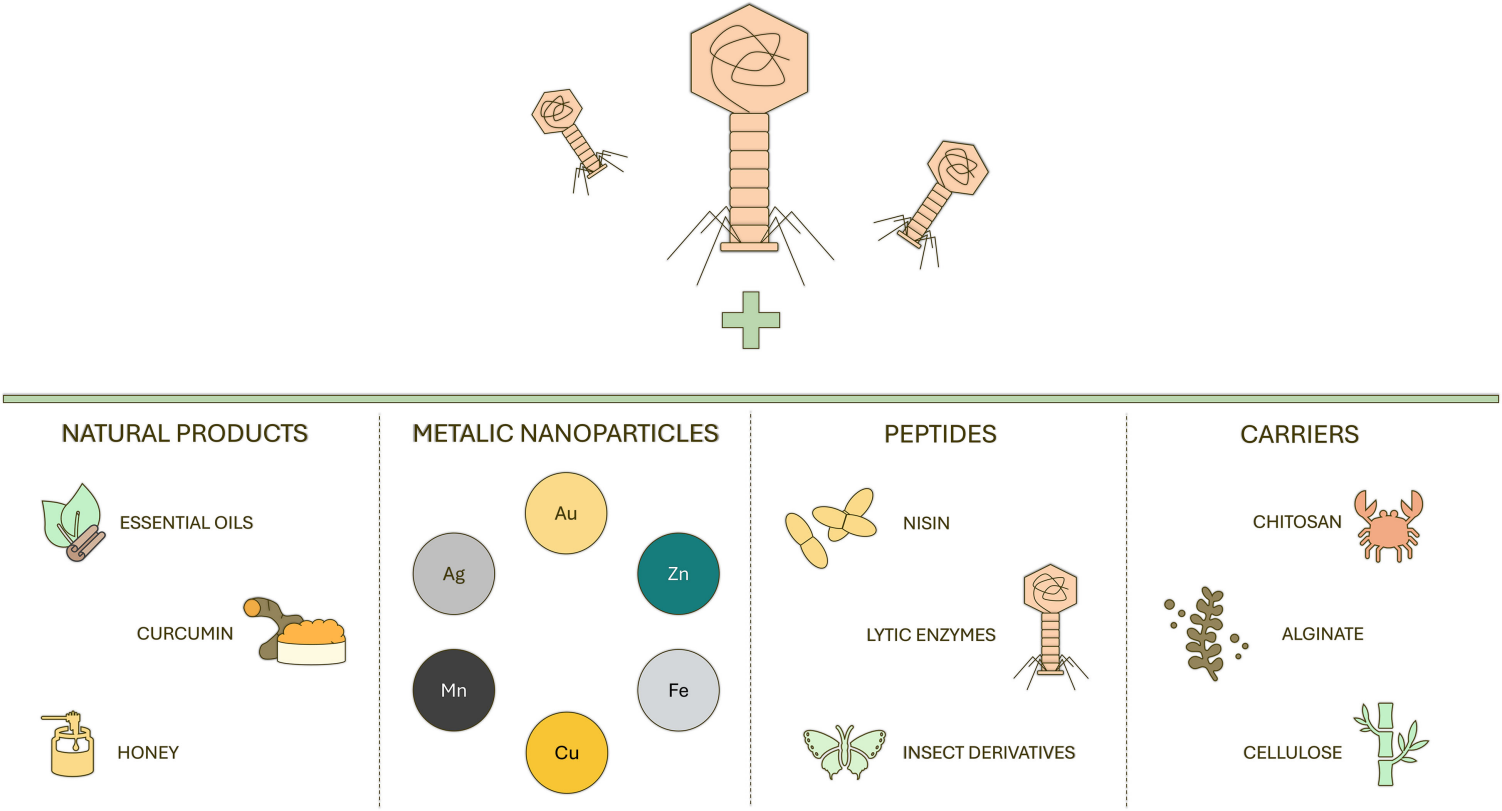
Schematic illustration of non-antibiotic antimicrobial classes used in combination with bacteriophages, including natural products, metallic nanoparticles, antimicrobial peptides, and biopolymer-based carriers. These agents enhance phage stability, delivery, and antibacterial activity through complementary physicochemical and biological mechanisms.

Therefore, rather than being seen as a universal solution, PAS provides a valuable conceptual framework for understanding how other antimicrobial agents can modulate phage activity. The mechanistic principles underlying PAS are not exclusive to antibiotics. These same principles can be observed when pairing phages with non-antibiotic antimicrobial compounds, enabling the exploration of alternative phage-based combination strategies (Lin et al., 2024; Wdowiak et al., 2025a; Janesomboon et al., 2025). Published reports show promising results for some of those combinations. However, they touch on the possible limitations or disadvantages as well. Explored combinations of phages with various antimicrobials for potential use in agriculture, food industry, and healthcare are presented in **Figure 3**.

## 4 Essential oils

Plant-derived antimicrobial compounds, particularly essential oils (EO’s), have attracted increasing attention as potential adjuvants for bacteriophage-based antimicrobial formulations. Essential oils are complex mixtures of volatile secondary metabolites, including terpenes, phenolics, and aldehydes, many of which exhibit broad-spectrum antibacterial activity (Bakkali et al., 2008; de Groot and Schmidt, 2016). Their antimicrobial effects include disruption of bacterial cell wall or outer membrane integrity, interference with metabolism, and induction of oxidative stress responses (Wang et al., 2020; Aljaafari et al., 2021). In the context of phage therapy, these properties are of particular interest because they target bacterial features that are directly relevant to phage infection. Alterations in cell wall and membrane structure, surface charge, and permeability can influence phage adsorption efficiency, while stress-induced physiological changes may affect intracellular phage replication dynamics (Addo et al., 2024; Leprince and Mahillon, 2023; Bruce et al., 2026). In addition, several essential oils and plant-derived compounds have been shown to weaken biofilm structure or increase bacterial susceptibility within biofilms, thereby facilitating phage biofilm penetration (Reichling, 2020; Mathlouthi et al., 2021; Rossi et al., 2022; Yang et al., 2025). Unlike antibiotics, EOs are commonly used in food preservation and agriculture and have a well-established regulatory status and high public acceptance (Matté et al., 2023; Ivanova et al., 2025). The research on the prospective use of phage-EOs combinations focuses mainly on food safety and agriculture (Matté et al., 2023; Stevanović et al., 2018). However, the complexity and variability of essential oil composition, as well as their potential to inactivate phages at higher concentrations, necessitate careful evaluation of synergistic interactions on a case-by-case basis (Sheng et al., 2016; Afshari and Rahimmalek, 2018; Kamarasu et al., 2025).

Multiple studies have examined the combined application of bacteriophages with essential oils or their individual components against both Gram-positive and Gram-negative bacteria (**Table 1**). Across diverse experimental systems, these combinations have generally resulted in greater bacterial reduction than either phages or essential oils alone, although the degree of synergy varied depending on bacterial species, phage type, and experimental conditions (Ghosh et al., 2016; Fokas et al., 2025). Reported synergistic effects were most frequently observed at subinhibitory concentrations, where membrane disruption and stress induction appeared to enhance phage adsorption and replication without compromising phage viability (Ebani and Mancianti, 2020; Kim et al., 2024; Fokas et al., 2025). Several studies demonstrated improved phage efficacy against bacterial biofilms following essential oil exposure, suggesting that partial biofilm disruption or increased bacterial cell wall permeability facilitated phage penetration (Han et al., 2024; Kao Godínez et al., 2025). In some cases, combined treatments resulted in significantly delayed emergence of phage-resistant bacteria (Maung et al., 2024; Elafify et al., 2025). Key findings and methodological parameters of published phage–essential oil synergy studies are summarized in **Table 1**.

**Table 1:** Studies on phage-essential oils synergy.

Phage	Essential oil or its compound	Bacterial species	Matrix	Key conditions	Main outcome	Phage resistance	Ref.
Phage K	alpha-pinene 3-carene	Cocktail of 4 *S. aureus* strains	Chicken breast	Reduction of bacterial load on the surface of raw chicken after exposure to 1.5% and 3.28% of EO compound and phage (MOI = 1)	No synergy detected	No data	(Ghosh, 2015)
	alpha-pinene 3-carene	4 *S. aureus* strains	Liquid culture	Growth Inhibition Assay after exposure to 1.5% and 3.28% of EO compound and phage (MOI = 1)	Observed synergy depended on temperature and host strain used	No data	(Ghosh et al., 2016)
BEC8	Trans-cinnamaldehyde (TC)	*E. coli O157:H7*	Organic baby spinach and baby romaine lettuce	Application of 0,5% TC and phage (10^6^ PFU) on top of the leaf spots previously inoculated with mixtures of the three Nal^R^*E. coli* O157:H7 strains	Complete inactivation of *E. coli* O157:H7 within 10 min and 1 hour on baby spinach leaves and baby romaine lettuce, respectively.	No data	(Viazis et al., 2011)
SALMONELEX™	Thymol Carvacrol	*S.* Typhimurium JWC-3001	Chicken	Sequential dipping of inoculated meat in phage stock (1.1×10^8^PFU/ml) and essential oils (1.6% w/v) for 3 minutes	Higher significant reduction (1.9 – 2.0 log CFU/g) than individual treatment (1.3 – 2.0 log CFU/g)	No data	(Moon et al., 2020)
vB_SauM_CP9	Thyme oil	*S. aureus* (MDR)	Chicken fillets	Reduction of bacterial load on the surface of raw chicken meat after exposure to 1% EO and phage (MOI = 10)	Higher significant reduction (87.22%) than individual treatment (56.67 – 76.94%)	No data	(Abdallah et al., 2021)
CAU-SEP-3	Thymol Geraniol	*Salmonella*	Biofilm on coupons (polypropylene and stainless steel) and quail eggs	Confocal Laser Scanning Microscopy (CLSM) and Computer Statistical Analysis Tool (COMSTAT) method	The synergistic effect observed at all surfaces also resulted in a reduction of bacterial virulence	No data	(Han et al., 2024)
LysPB32 (lysin)	Allyl isothiocyanate Carvacrol Eugenol Thymol	*S.* Typhimurium	Liquid culture	Time-kill curve assay after exposure to EO and lysin (100 μg/mL)	Reduction of bacterial cells to below the detection limit (20 CFU/ml) after 12 h incubation at 37 °C	No data	(Kim et al., 2024)
			Cooked ground beef	Bacterial growth inhibition test	Reduction of bacterial cells by over 2 logs after 24 h of storage at 37 °C and 7 days of refrigerated storage		
vB_LmoS-PLM9	Cinnamon bark Cinnamon cassia	*L. monocytogenes* 193	Liquid culture	Bacterial count after exposure 0.02-0.03% of EO and phage at MOI = 10	Synergistic effect without regrowth observed after 24 h with 0.03% of EO	Regrow observed only in liquid culture with low EO concentration and phage or EO alone	(Maung et al., 2024)
			Pasteurized milk	Bacterial count after exposure 0.125% of EO and phage at MOI = 100	A synergistic effect without regrowth was observed after 24 h at all combinations		
			Smoked salmon	Bacterial count after exposure to 0.125 – 0.25% of EO and phage at MOI = 1000	No synergy detected		
MS2 + T7 phages	Cinnamon Thymol	*E. coli* ATCC 15597	Liquid culture	Time-kill curve assay at 37 °C after exposure to ½ MIC of EO and phage (MOI = 0.1)	Lytic activity of the cocktail significantly enhanced in the presence of all EOs after 24-h incubation at 37 °C	Less phage resistant mutants (6-8%) than in phage alone samples (88%)	(Elafify et al., 2025)
phiLLS	Oregano oil	*E. coli* O157:H7 *E. coli* BALL 1119 *Salmonella* spp. *L. monocytogenes* *S. aureus* *B. cereus*	Liquid culture	Bacterial growth after addition of EO (2 mg/ml) and phage (MOI = 1) to culture with OD_600_ = 0.1 for 9 h	Synergistic effect only for *E. coli* (a Bliss synergy index of 10.8% at 9 h)	No data	(Kao Godínez et al., 2025)
			96-well plate biofilm	Reduction after 24 h of incubation with EO (2 mg/ml) and phage (MOI = 1)	Synergy in biofilm biomass reduction for *L. monocytogenes*, *S. aureus*, and *Salmonella* *S. aureus* exhibited synergy in metabolic inactivation (TTC) Antagonistic effect for *B. cereus*		

Despite these promising findings, important limitations remain. Essential oils exhibit substantial variability in chemical composition depending on plant species, extraction method, and formulation, complicating reproducibility and standardization (Afshari and Rahimmalek, 2018; Talebi et al., 2024). Concentration-dependent effects should not be overlooked, as doses sufficient to damage bacterial membranes may also inactivate phage particles or reduce their stability (Sheng et al., 2016; Kamarasu et al., 2025). Mechanistic insights are often inferred rather than directly demonstrated, underscoring the need for integrated approaches combining microbiological, biophysical, and omics-based analyses (Fokas et al., 2025). Addressing these gaps will be essential for determining whether essential oils can serve as reliable and scalable adjuvants to phage-based antimicrobial strategies.

## 5 Bacteriocins

Bacteriocins are ribosomally synthesized antimicrobial peptides or proteins produced by bacteria that exhibit potent and often narrow-spectrum activity against closely related species (EFSA Panel on Food Additives et al., 2017; Rendueles et al., 2022). Their antibacterial effects commonly involve pore formation in the cytoplasmic membrane, inhibition of cell wall biosynthesis, or interference with essential intracellular processes (Vasilchenko and Valyshev, 2019; Telhig et al., 2020; Martínez et al., 2020; Rendueles et al., 2022). Due to their defined molecular targets and proteinaceous nature, bacteriocins have been explored as alternatives or complements to conventional antibiotics in both clinical and food-related applications (Rendueles et al., 2022; Leprince and Mahillon, 2023; Fokas et al., 2025). From a phage-therapy perspective, bacteriocins are seen as promising combination partners as their mechanisms of action overlap. Unlike essential oils, bacteriocins are chemically well-defined and can be produced using standardized methods, improving reproducibility and dosing precision. However, their relatively narrow spectrum of activity and susceptibility to proteolytic degradation present challenges for therapeutic deployment, particularly *in vivo*.

Several studies have investigated the combined use of bacteriophages and bacteriocins against clinically and industrially relevant bacterial pathogens, including *Staphylococcus aureus, Listeria monocytogenes*, and *Salmonella* sp. (**Table 2**). Research on phage–bacteriocin synergy has focused primarily on *L. monocytogenes*, with nisin as the most studied compound, often combined with commercial phage products such as P100 or LMP-102 (Rendueles et al., 2022). Subsequent studies demonstrated enhanced reductions in bacterial load across diverse food matrices, including fruit, fish, meat, and dairy products, frequently outperforming individual treatments and chemical sanitizers (Dykes and Moorhead, 2002; Leverentz et al., 2003; Soni et al., 2014; Figueiredo and Almeida, 2017; Lewis et al., 2019; Ibarra-Sánchez et al., 2018; Baños et al., 2016a; Rodríguez-Rubio et al., 2015; Komora et al., 2020) Similar synergistic effects have been reported against *S. aureus*, where combinations of nisin with phages or phage-derived endolysins showed enhanced efficacy in milk and broth, particularly at sub-optimal concentrations, despite matrix-dependent outcomes, and without cross-resistance between phages and bacteriocins (Martínez et al., 2020; Gembara and Dąbrowska, 2024; Duc et al., 2020a; Kim et al., 2019). Beyond these pathogens, phage–bacteriocin combinations improved control of *Salmonella* sp. in food and biofilm models and achieved complete eradication of *Clostridium perfringens* in co-culture (Wang et al., 2017; Yüksel et al., 2018; Heo et al., 2018). A detailed summary of bacterial targets, phages, bacteriocins, experimental conditions, and outcomes is provided in **Table 2**.

**Table 2:** Summary of studies focusing on phage-bacteriocin synergy.

Phage	Bacteriocin	Bacterial species	Matrix	Key conditions	Main outcome of the combined application	Resistance after combined treatment	Ref.
LH7	Nisaplin^®^ (*L. lactis*)	*L. monocytogenes* L62 and L99	Liquid culture	Growth inhibition after exposure to nisin (5×10^3^ IU ml^−1^) and phage (3×10^3^ pfu ml^−1^) at 7°C and 30 °C	The synergistic effect depends on the strain, temperature, and culture phase	No regrowth observed at the end of the experiment (day 22)	(Dykes and Moorhead, 2002)
			Meat	Growth inhibition after rinse of contaminated meat cubes in combination of nisin (5×10^3^ I/ml) and phage (3×10^3^ PFU/ml) stored at 7°C and 30 °C	Higher decrease (by 1.6 log CFU) than for nisin (by 1 log) and phage (no effect) alone	No changes in bacterial level after initial decrease	
LM-103 and LMP-102 cocktails	Nisaplin^®^ (*L. lactis*)	*L. monocytogenes*	apples and honeydew melons	Growth inhibition on fruit slice surface after exposure to nisin (1, 200, and 400 IU/25 μl) and phage (10^7^ PFU/ml) at 10°C	On both fruits, the combination reduced the bacterial populations more than nisin alone	No data	(Leverentz et al., 2003)
P100	Nisin N5764	Mix of 5 *L. monocytogenes* strains	cold-smoked salmon	Growth inhibition on food cubes after exposure to nisin (500 ppm) and phage (10^8^ PFU/cm^2^)	Reduction to an undetectable level in 24 h	No data	(Soni et al., 2014)
FWLLm1 and FWLLm3	coagulin C23	*L. monocytogenes* 2000/47 strain	Liquid culture	Growth inhibition after exposure to coagulin (584 AU/ml) and phage (MOI = 10)	Synergistic effect observed with the decrease under the detectable level in 6 h for FWLLm3 in liquid and FWLLm1 in milk	Combination reduced phage-resistant ratio from 50% to 0% for FWLLm3	(Rodríguez-Rubio et al., 2015)
			Milk	Growth inhibition after exposure to coagulin (584 AU/ml) and phage (MOI = 10 for FWLLm3 and 100 for FWLLm1)			
P100	enterocin AS-48	*L. monocytogenes*	Raw hake and salmon Smoked salmon	Growth inhibition on food slices after exposure to bacteriocin (0.37 μg/cm^2^) and phage (2.3 × 10^7^ PFU/cm^2^)	In raw fish, a combined treatment reduced listeria below detection levels up to 7 days, while in smoked salmon, up to 15 days	No data	(Baños et al., 2016a)
P100	Nisin N5764	*L. monocytogenes*	Ready-to-eat pork ham	Growth inhibition on food slices after exposure to nisin (0.0012 μg/g) and phage (5 × 10^5^ PFU/g)	The combination had a small anti-listeria effect at zero h, but almost 3 log reduction was observed at 72 h	No data	(Figueiredo and Almeida, 2017)
P100	Nisaplin^®^ (*L. lactis*)	*L. monocytogenes* ScottA	Liquid culture and Coleslaw liquid fraction	Checkerboard assay with bacteriocin (0 - 1600 µg/ml) and phage (MOI = 0 – 100)	No synergistic effect in liquid culture	No data	(Lewis et al., 2019)
			Coleslaw	Growth inhibition after exposure to bacteriocin (25 µg/1g of food) and phage (MOI = 2.5, 25, 50) at 4°C	Higher activity of the combination than bacteriocin alone, but not than phage alone	No resistance to bacteriocin or phage was detected after 10 days	
Listex™ P100	pediocin PA-1	*L. monocytogenes* Scott A and Lm 1751	milk	Growth inhibition after exposure to coagulin (584 AU/ml) and phage (MOI = 10 for FWLLm3 and MOI = 100 for FWLLm1)	Inoculum-dependent synergy was observed at three and seven days of storage for Lm 1751 and only immediately after treatment for Lm Scott A	Regrowth observed in FWLLm3-treated samples	(Komora et al., 2020)
Φ35 and Φ88	nisin	*S. aureus*	liquid culture	Checkerboard assay with nisin (1.56 to 0.06 µg/ml) and phage (3 × 10^5^ to 3 × 10–^3^ PFU/ml)	The combination resulted in a survival of *S. aureus* that was > 1 log unit less than in antimicrobial samples alone samples	Nisin adapted strain resulted in partial cross-resistance with phages, while phage-resistant mutants remained sensitive to nisin	(Martínez et al., 2008)
			milk	Growth inhibition after exposure to nisin (1.5 µg/ml) and phage cocktail (10^3^ PFU/ml)			
LysH5 lysin	nisin	*S. aureus*	liquid culture	Checkerboard assay with nisin (0.75 μg/ml to 0.00075 μg/ml) and endolysin (50 U/ml to 0.78 U/ml)	The combination resulted in up to a 64-fold and 16-fold reduction of the nisin and endolysin MICs, respectively	No resistance to phage was detected even in nisin-adapted mutants	(García et al., 2010a)
			pasteurized milk	Growth inhibition after exposure to nisin (1.5 µg/ml) and endolysin (10^3^ PFU/ml)	The combination resulted in a complete clearance after 6 h of incubation		
SA46- CTH2	nisin	*S. aureus*	liquid culture and biofilm	Growth inhibition after exposure to nisin (100 IU/ml and 10 IU/ml) and phage (10^9^ PFU/ml and 10^8^ PFU/ml)	A combination of higher concentrations resulted in the complete eradication of bacteria	No data	(Duc et al., 2020a)
			Biofilm in 96-well plate and on stainless steel coupons (SSC)	Biofilms treated with phage (10^10^ PFU/ml) and nisin (100–1000 IU/mL for plate and 10–100 IU/mL for SSC)	No synergistic effect observed		
			pasteurized milk	Growth inhibition after exposure to nisin (100 IU/ml) and phage (10^9^ PFU/ml) at 4°C	No synergistic effect observed		
SA46- CTH2	nisin	*S. aureus*	liquid culture and biofilm	Growth inhibition after exposure to nisin (100 IU/ml and 10 IU/ml) and phage (10^9^ PFU/ml and 10^8^ PFU/ml)	A combination at higher concentrations resulted in the complete eradication of bacteria	No data	(Duc et al., 2020a)
			Biofilm in a 96-well plate and on stainless steel coupons (SSC)	Biofilms treated with phage (10^10^ PFU/ml) and nisin (100–1000 IU/mL for plate and 10–100 IU/mL for SSC)	No synergistic effect observed		
SAP84	crude bacteriocin (*L. lactis*)	*S. aureus*	Liquid culture	Growth inhibiton after exposure to bacteriocin (12.5, 25, 50, or 100 AU/ml) and phage (MOI = 0.1)	Synergistic inhibition with 50 or 100 AU/mL of the bacteriocin	No data	(Kim et al., 2019)
phiIPLA-RODI and LysRODIΔAmi lysin	nisin	*S. aureus*	Laboratory-scale cheeses	Treatments were added during cheese coagulation: nisin (1.5 μg/ml), LysRODIΔAmi (0.6 and 0.12 μM), and phage (10^7^ PFU/ml)	After storage, all the combinations led to elimination of bacterial contamination below the detection level.	No data	(Youssef et al., 2023)
fmb-p1	nisin	*S.* Typhimurium	fresh chilled pork	Growth inhibition on fruit slice surface after exposure to combinations of 5% nisin, phage (10^10^ PFU/ml), and potassium sorbate	The significant bactericidal effect was only visible in combinations with phages	No data	(Wang et al., 2017)

Overall, available evidence suggests that synergy between bacteriophages and bacteriocins (similarly to phage-EOs synergy) is most commonly associated with membrane and cell wall perturbations that enhance phage adsorption and genome delivery, as well as through stress-induced physiological changes that favor phage replication (Vasilchenko and Valyshev, 2019; Telhig et al., 2020; Martínez et al., 2020; Rendueles et al., 2022). Nevertheless, several limitations constrain the broader application. Many bacteriocins display narrow target specificity, which may restrict their utility against diverse bacterial populations. Proteolytic instability, potential immunogenicity, and reduced activity in complex biological matrices are additional challenges, particularly for systemic applications. Moreover, most studies have been conducted *in vitro*, with limited assessment of pharmacokinetics, safety, and *in vivo* long-term resistance dynamics. Addressing these knowledge gaps will be essential for determining whether bacteriocins can serve as adjuvants to phage-based antimicrobial strategies.

## 6 Nanoparticles

Nanoparticles (NPs), including metallic, metal oxide, and polymer-based nanomaterials, have emerged as promising antimicrobial agents due to their high surface area, tunable physicochemical properties, and diverse mechanisms of antibacterial action. Antimicrobial nanoparticles, such as silver, gold, zinc oxide, copper oxide, and chitosan-based materials, exhibit bactericidal effects by disrupting membranes, generating reactive oxygen species, interfering with metabolic pathways, and damaging nucleic acids and proteins.

In the context of phage therapy, nanoparticles are seen as promising not only as direct antimicrobial agents but also as enhancers of phage activity or delivery systems. Nanoparticles can both alter bacterial surface properties, allowing for more efficient phage adsorption, or act as carriers that protect phages from unfavorable environmental conditions. A growing number of studies have explored the combined use of bacteriophages with antimicrobial nanoparticles against a range of bacterial pathogens, including *E. coli, Pseudomonas aeruginosa*, and *S. aureus* (**Table 3**). Sub-MIC concentrations of biogenic AgNPs combined with *Salmonella* phages (ZCSE2, ZCSE6, P22) significantly enhanced bacterial inhibition in broth and food models without phage inactivation or increased cytotoxicity, even at low MOIs, and consistently outperformed individual treatments (Abdelsattar et al., 2021; 2022; Makky et al., 2023; Wdowiak et al., 2025a). Genetic modification of phages to display AgNP-binding peptides further improved antibiofilm efficacy at high phage titers while maintaining stability and safety for eukaryotic cells (Szymczak et al., 2024; Szymczak and Golec, 2024a). Comparable synergistic effects were observed against *S. aureus*, including biofilm dispersal and reduced survival rates, as well as with phage-mimicking silver–gold nanostructures active against MRSA (Hopf et al., 2019; Manoharadas et al., 2021; Wdowiak et al., 2025a). Although AgNPs dominate current research, synergy has also been demonstrated with gold, copper, and zinc nanoparticles (Hopf et al., 2019; Peng et al., 2020; Alipour-Khezri et al., 2024; Zhang et al., 2024a).

**Table 3:** Summary of key studies on phage-nanoparticle synergy.

Phage	Nanoparticle metal	Bacterial species	Matrix	Key conditions	Main outcome of combined application	Resistance after combined treatment	Ref.
ZCSE6	silver	*S.* Typhimurium	Liquid culture	Time killing curve after exposure to particle (11.25 and 5.7 μg/ml) and phage (MOI = 0.1)	Synergistic effect depends on nanoparticle concentration	No data	(Makky et al., 2023)
ZCSE2	silver	*S. enterica* (MDR)	Liquid culture	Time killing curve after exposure to particle (10 μg/ml) and phage (MOI = 0.1)	Synergistic effect observed when compared with the same concentrations of antimicrobials alone	No regrow of culture when phage was combined with nanoparticles	(Abdelsattar et al., 2021)
T7	silver	*E. coli*	Biofilm in 96-well plate	Crystal violet biofilm analysis after exposure to phage binding metal particles - T7Ag-XII-AgNPs and different Ag concentration	T7Ag-XII-AgNPs biomaterial is significantly more effective over a longer period than phages or nanomaterials alone, even at lower doses.	No data	(Szymczak et al., 2024; Szymczak et al., 2024)
P22 and vB_SauS_CS1	green tea extract-capped silver (G-TeaNPs)	*S. aureus* *S. enterica*	Liquid culture	Growth inhibition after exposure to G-TeaNPs (0.1 to 0.0001 mg/ml) and phage (1, 10, and 100) for 3 h	All combinations with MOI = 10 and 100 were more active than antimicrobials alone, with efficiency depending on the strain and used concentration.	No data	(Wdowiak et al., 2025b)
ϕ44AHJD	silver	*S. aureus*	Glass cover slip	Live/dead assay after submersion of slip with 168h-biofilm in the inhibitory concentration of AgNP (1 mM) and phage (1 × 10^8^ PFU ml^−1^)	Synergistic effect observed both after 1 and 18 h of treatment, while phages alone did not reduce the biofilm after 1h, and the efficacy of AgNP was the same at both times.	Viable phage-resistant cells were observed in the sample treated with phage only	(Manoharadas et al., 2021)
PB10 and PA19	zinc (ZnO-NP)	*P. aeruginosa*	Liquid culture	Biofilm formation kinetics assay after treatment of liquid culture with nanoparticle (500 μg/ml) and phage (6 × 10^6^)	Only samples with PA19 showed more efficient biofilm reduction than particles alone.	No data	(Alipour-Khezri et al., 2024)
			Biofilm	Biofilm reduction crystal violet assay after treatment of liquid culture with nanoparticle (500 μg/ml) and phage (10^8^ PFU/ml)	All combinations with phages were more active then phages or nanoparticles alone after 48 h.		
ϕPB2	copper (CuONPs)	*R. solanacearum*	Liquid culture	Growth inhibition assay with plate counting method after exposure to nanoparticle (250 mg/L) and phage (10^3^, 10^4^, 10^5^, 10^6^, 10^7^ PFU/ml)	Positive correlation with the best activity at 10^6^ and 10^7^ PFU/mL	No data	(Zhang and Ahn, 2025)
M13-g3p(If1), M13-g3p(Pf1), M13-g3p(ϕLf), M13-g3p(ϕXv), and M13-g3p(CTXϕ)	gold	*E. coli* *V. cholerae* *X. campestris* *P. aeruginosa*	Liquid culture	Photothermal ablation of bacterial cells treated with metal-carrying phages (10^11^ PFU/ml) with NIR laser	Attached to phage gold nanorods, which release energy, locally generating heat that efficiently kills targeted bacterial cells	No data	(Peng et al., 2020)
			Biofilm (only *P. aeruginosa*)	Photothermal ablation of biofilm cells treated with metal-carrying phages (10^13^ PFU/ml) with NIR laser			

A detailed overview of bacterial targets, phages, nanoparticle types, physicochemical properties, experimental conditions, and observed effects is provided in **Table 3**.

Taken together, existing studies indicate that synergy between bacteriophages and nanoparticles most commonly arises from indirect enhancement of phage activity rather than simple additive antibacterial effects. In some applications, nanoparticles have also been used as carriers or protective matrices to improve phage stability and persistence under environmental stress (Peng et al., 2020).

Despite these promising results, significant challenges limit the translational potential. Many NPs exhibit dose-dependent cytotoxicity toward eukaryotic cells and may accumulate in environmental or biological systems, raising safety and ecological concerns (Xiong et al., 2022; Islam, 2025). Regulatory frameworks governing nanoparticle use in health, food industry, or agriculture are not yet fully defined, while large-scale manufacturing with consistent quality and reproducibility presents an additional challenge (Wang et al., 2024; Wdowiak et al., 2025a). Future research should prioritize systematic optimization of nanoparticle properties, rigorous safety assessment, and mechanistic studies that distinguish true synergy from concentration-dependent additive effects.

## 7 Other antimicrobials

In addition to essential oils, bacteriocins, and nanoparticles, a range of other non-antibiotic compounds have been investigated as adjuncts to bacteriophage-based antimicrobial strategies. Although these agents differ substantially in origin and primary function, they share the capacity to modulate bacterial physiology, alter environmental conditions, or improve phage stability and delivery. This section summarizes selected examples that do not fall into the preceding categories but provide insight into additional mechanisms by which phage efficacy may be enhanced.

### 7.1 Flavonoids

Flavonoids and other plant polyphenols can enhance the efficacy of bacteriophages against drug-resistant bacterial strains. Pimchan et al. tested combinations of bacteriophages with crude plant extracts against *E. coli*. They observed that phages alone or in combination with extracts reduced bacterial counts by ~2–3 log_10_ within 6 h, but the effect was short-lived. After 24 h, there was no difference between the treatments. Furthermore, some of the extracts showed anti-phage activity at inhibitory concentrations, emphasizing the importance of characterizing plant extracts thoroughly before combining with phage therapies (Pimchan et al., 2018). However, more recent studies reported more promising results. Chen-Yu et al. showed that the flavonoids myricetin and quercetin increased E. coli susceptibility to lytic phage infection by downregulating chaperone genes, such as dnaK, and weakening bacterial stress responses (Lin et al., 2024). Similarly, Janesomboon et al. found that combining curcumin with a phage targeting multidrug-resistant *Acinetobacter baumannii* achieved rapid, sustained bacterial clearance compared to either treatment alone (Janesomboon et al., 2025).

### 7.2 Host-derived antimicrobials

Lactoferrin, a component of the innate immune system in mammals, came into the spotlight during the COVID-19 pandemic and has shown antibacterial and antiviral activity (Ammons and Copié 2013; Bolat et al., 2022). Its antibacterial effect comes from iron sequestration, enzymatic degradation of peptidoglycan, or disruption of bacterial membranes, thereby targeting processes relevant to phage infection (Aguila et al., 2001; Kim et al., 2025). In several studies, co-application of phages with host-derived antimicrobials resulted in greater bacterial reduction than monotherapies, increasing antibiofilm activity, phage plating efficacy, and adsorption rate (Kosznik-Kwaśnicka et al., 2022; Grzenkowicz et al., 2025). Studies in murine models also demonstrated Lf’s positive effect on the outcome of phage treatment (Zimecki et al., 2004). Proposed mechanisms underlying phage-lactoferrin synergy include improved phage access to bacterial cells due to weakened cell walls, as well as altered bacterial stress responses that favor phage replication. However, reported outcomes remain highly context-dependent, with efficacy influenced by protein concentration, bacterial species, and environmental conditions (Grzenkowicz et al., 2025).

### 7.3 Environmental and physicochemical modulators

Several studies have examined compounds that lack vigorous intrinsic antibacterial activity but modulate physicochemical conditions that influence phage stability and delivery. These include UV-protective agents, pH-adjusting compounds, and mucoactive substances. The studies have shown that these agents can enhance phage persistence or diffusion in complex environments, such as mucus, food matrices, or plant surfaces. Choudhary et al. observed that incorporating UV-protectants into phage formulations significantly improved phage persistence and infectivity on plant surfaces, enhancing biocontrol performance under sunlight exposure (Choudhary et al., 2023). Another study revealed that mucoactive drugs, such as N-acetylcysteine and ambroxol, synergistically increased phage activity against Pseudomonas aeruginosa and Klebsiella pneumoniae by altering bacterial surface properties and mucus composition (Sui et al., 2025). In such systems, improved phage survival or spatial distribution translated into enhanced antibacterial efficacy, even in the absence of direct antimicrobial activity from the adjunct compound (Choudhary et al., 2023; Sui et al., 2025). These findings highlight that functional enhancement of phage delivery and stability can be as important as direct bacterial killing in determining treatment success.

Collectively, studies involving these compounds demonstrate that phage efficacy can be enhanced not only through direct antimicrobial synergy but also through modulation of bacterial physiology, environmental conditions, and phage stability. Reported outcomes varied substantially across systems, reflecting differences in compound class, bacterial targets, experimental matrices, and treatment protocols (Grzenkowicz et al., 2025; Sui et al., 2025).

## 8 Summary

The growing burden of MDR bacterial infections has renewed interest in bacteriophage-based antimicrobials as alternatives or complements to conventional antibiotics. However, the efficacy of phage therapy as a stand-alone approach is constrained by factors including narrow host range, resistance development, delivery challenges, and context-dependent activity in complex biological and environmental matrices. These limitations have driven increasing attention toward combination strategies designed to enhance phage performance.

The evidence summarized in this review demonstrates that non-antibiotic antimicrobial compounds can act as effective phage adjuvants across diverse application areas, including clinical therapy, food safety, and agriculture. Essential oils, bacteriocins, nanoparticles, and other agents have been shown to enhance phage efficacy through multiple, often overlapping, mechanisms. These include: destabilization of bacterial cell walls and outer membrane integrity, which facilitates phage adsorption; modulation of bacterial stress responses that influence phage replication dynamics; disruption of biofilm structure, which facilitates phage penetration; and improvement of phage stability or delivery under adverse environmental conditions. Importantly, in several systems, these effects have also been shown to delay the emergence of phage-resistant bacterial clones, highlighting the potential of combination strategies to constrain the phage-bacteria arms race.

At the same time, the studies show that phage–adjuvant synergy is highly context-dependent. Outcomes vary depending on bacterial species and strain, phage type, compound class, concentration, treatment time, and order of administration. In some cases, non-antibiotic compounds reduced phage stability or infectivity at higher concentrations, emphasizing the need for careful optimization and mechanistic compatibility. Moreover, to date, there is limited data from *in vivo* models, as most of the studies discussed in this review focused on *in vitro* experiments. Therefore, the translation of these findings into practice remains difficult to assess.

Future research should focus on mechanistic approaches to phage-based combination therapy. Systematic studies on dose–response relationships, timing, and resistance are needed to identify actual synergistic effects from additive or antagonistic ones. Combining microbiological tests with imaging, biophysical analysis, and omics methods will be crucial to understanding the molecular and physiological basis of these synergies. Additionally, more attention should be paid to safety, regulatory issues, and ecological impacts, especially for nanoparticle systems and environmental applications.

In conclusion, non-antibiotic antimicrobial compounds represent a diverse and promising tool for enhancing bacteriophage efficacy across multiple sectors. However, based on the current data, these agents should be viewed as context-specific modulators of phage activity, rather than serving as universal solutions. Continued interdisciplinary efforts will be critical to translate phage–adjuvant combination strategies from experimental proof-of-concept studies into robust, safe, and scalable antimicrobial interventions.
